# The Role of Patient Support Groups in Neuroendocrine Neoplasms

**DOI:** 10.1007/s11912-021-01046-6

**Published:** 2021-03-22

**Authors:** Teodora Kolarova, Catherine Bouvier

**Affiliations:** 1 International Neuroendocrine Cancer Alliance, Boston, MA USA; 2 Neuroendocrine Cancer UK, Leamington Spa, UK

**Keywords:** Neuroendocrine cancer, Evidence, Change, Role, Empowerment, Advocacy

## Abstract

**Purpose of Review:**

The purpose of this review is to establish the role patient support groups play in NENs.

**Recent Findings:**

Published data on the role and work done by these groups is extremely sparse, so the review references publications in the wider cancer advocacy context. For the purposes of the review, a survey was carried out among the members of a global umbrella organization to ascertain the level of activities undertaken in support of the NEN patient community.

**Summary:**

The concept of “support groups” has changed significantly, as these groups have evolved from patient peer-to-peer support provision to a strategic focus on improving awareness and education among all stakeholders, generating patient evidence to influence policies for access to optimal diagnostics, treatment, and care, and setting the research agenda. Today, NEN patient organizations have an instrumental role of catalysts of change across the healthcare spectrum—especially relevant in a setting of less common and not well-understood diseases, where clear pathways and guidelines are still a challenge.

## Introduction

### Neuroendocrine Neoplasms

Neuroendocrine neoplasms are rare neoplasms arising from neuroendocrine cells [1]. The extensive presence of these specialized cells in the body facilitates occurrence in many organ systems, primarily the gastrointestinal tract, pancreas, and lung [1, 2]. The body-wide presentation, diverse and potentially nonspecific symptoms, and the lack of education among the wider healthcare professional community make timely diagnosis and management challenging [1, 3].

There has however been a recent re-classification of neuroendocrine neoplasms, which reflects important advancements in our understanding of tumors of the digestive system. For the first time, certain tumor types are defined as much by their molecular phenotype as their histological characteristics; however, in most instances, histopathological classification remains the gold standard for diagnosis [4].

Reported incidence of NENs varies greatly, likely owing to underreporting and varying nomenclature/classifications [5]. Recent data suggest an incidence of 6.98 per 100,000 in the USA and 8.6 per 100,000 in the UK [6, 7•]. Incidence rates have been increasing, possibly due to increased NEN awareness and improved diagnostic tools [6, 7•].

### The Role of “Support Groups”

“Support groups” across the globe have advanced and matured, and for many, the support service is part of a much wider remit of activity. In this current era, “advocacy” organizations also play an integral navigational role for patients and their families, alongside providing significant healthcare support, patient peer-to-peer support, training, education, and awareness, while undertaking vital data collection with reference to real-life lived experience.

However, there is such a sparsity of published data on the role and work done by these groups in the context of NENs, that the only acceptable references come from the advocacy organizations working in other cancer types. This only highlights the lack of focus on the rare and uncommon cancer fields, and this issue needs to be addressed.

“People with a rare or less common cancer are disadvantaged at every step of their journey with cancer. From the speed of diagnosis through to treatment and research, people with rare and less common cancers often get a second-class service and a poor deal. It is time to level the playing field - by ensuring that people are treated equally, that they get the specialist treatment they need and that they are properly supported to live their lives as fully as possible. It is time for policy makers, health professionals and commissioners to acknowledge the differences in patient experience and to take positive and meaningful steps to address them.” [8]

“Rare cancer patient advocates are well aware of just what these unmet needs are. For example, there is inequity in accessing promising new therapies; a paucity of specialists to treat rare cancers; incomplete and inconsistent registries; not enough clinical trials; and a lack of sufficient research funding, to name just a few of the challenges. Additionally, people with rare cancers can be misdiagnosed or diagnosed very late, and they face huge uncertainties about treatment decisions. They can also struggle with a lack of information and support due to the uncommon nature of their disease.” [9]

## Current State of Play

There is much a newly diagnosed NEN patient has to contend with—a sense of being invisible, surrounded by the unfamiliar especially around diagnostics and treatments, not conforming to the “treatment, recovery, and survivorship/decline” model of cancer care, coping with uncertainty and ultimately having to live with cancer. This involves a different way of thinking about cancer to the one most are used to. It means learning to live with cancer as part of your everyday life: being vigilant, assertive, and knowledgeable. The community services provided by advocacy organizations globally have key missions to support the day-to-day “living with” this disease and the burden that lived experience brings.

“When I was first diagnosed with neuroendocrine cancer, finding the neuroendocrine cancer community was like being pulled to safety by the lifeboat crew when being lost in a storm. Not only did they understand the fears generated by the complexity of my disease, they have provided continual help and support, to help me navigate my way through all the twists and turns generated by my journey.”, Kath Lewis, Patient Ambassador, Neuroendocrine Cancer UK.

The quote above clearly shows that the role of patient advocacy organizations is indeed instrumental, and the support they provide at every level does make a difference for NEN patients.

**Fig. 1:**
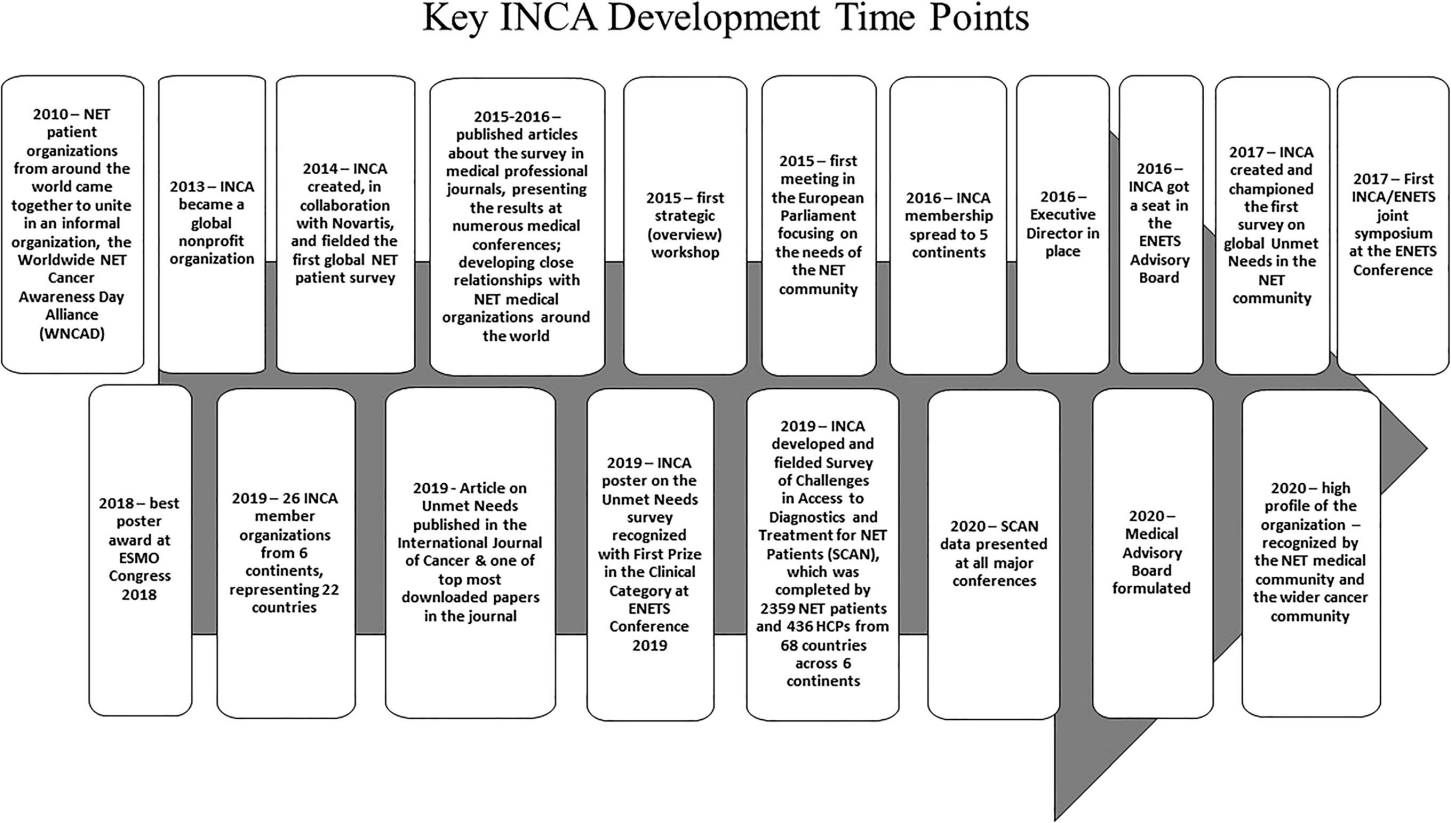
The development curve of the International Neuroendocrine Cancer Alliance (INCA)

As a global organization, the International Neuroendocrine Cancer Alliance (INCA) is committed to be the global voice of NEN patients and, like the whole advocacy movement, has multiple aspects to its work. As an umbrella patient organization, representing 26 countries on six continents, INCA’s major focus lies in creating platforms for collaboration with the key stakeholders in the community to raise awareness about NENs, improve access to optimal care and treatment for all NEN patients, and facilitate meaningful involvement of patients in research. INCA has concentrated its efforts on identifying and addressing the unmet needs of the NEN patient community in an attempt to improve the lives of all those affected by this complex disease, no matter where in the world they live (Fig. 1).

In July 2020, INCA carried out a survey among its membership using SurveyMonkey® to ascertain the level of activities undertaken in supporting the NEN patient community. Twenty out of 26 members responded to the survey, and of those participants, 94% were solely dedicated to the care of the NEN community, as opposed to a more general cancer organization supporting multiple cancer types

Of the organizations, coming from varied background in terms of years of existence—ranging from 2 to 52 years, 95% reported to be involved in providing information via their social media channels and collaborating with other advocacy organizations as their key activities. Eighty-five percent focused on organizing trainings, educational forums, information days for NEN patients and carers, and national and international cancer awareness/education campaigns, and conducting surveys among their community to monitor quality of diagnostics, treatment, and care to identify unmet needs. In addition, 80% said they supported help line for NEN patients, and the same percentage provide information about clinical trials (via website; newsletter, others) and collaborate with all relevant stakeholders to drive change and gather data.

Table 1 clearly defines 29 activities, with over 75% of organizations engaging in support services, education events, campaign work, data collection, patient information production, and collaborative projects—all with the support of a dedicated medical advisory board. Over 60% are involved in awareness raising, provide local or national support groups, present at conferences and events, and utilize social media to highlight the needs of the community, and indeed to develop that very community ethos.

**Table 1:** The role of NEN patient advocacy groups

Advocacy group activity	
Provide information via your organization’s social media channels (Facebook, Twitter, Instagram)	95%
Collaborate with other advocacy organizations	95%
Organize trainings, educational forums, info days for NEN patients and carers	85%
Involvement in National & International cancer awareness and education campaigns	85%
Conduct surveys among your community to monitor quality of diagnostics, treatment and care and to identify unmet needs	85%
Support help line for NEN patients	80%
Provide information about clinical trials (via website; newsletter, others)	80%
Collaborate with all relevant stakeholders to drive change and gather data	80%
Have a dedicated medical advisory board	75%
Work to improve access to optimal diagnostics, treatment and care	75%
Run social media awareness campaigns	70%
Speak with community groups: giving public talks to general audiences or healthcare professionals	65%
Coordinate support groups (incl. virtual)	60%
Raise awareness and educate about NENs via local media (TV, radio, press)	60%
Provide merchandise goods to raise awareness about NENs	50%
Produce reports and publications to promote the results from monitoring surveys	50%
Organize fundraisers for NET research or raise funds for research	50%
Organize public events to raise awareness about NENs	45%
Provide awards to medical researchers directly	40%
Run advocacy campaigns aimed at policy-makers	40%
Participate in research design—be involved in setting research priorities, approving protocols, patient information	35%
Donate research grants	35%
Recruit patients for clinical trials	30%
Testify at governmental hearings, incl. involvement in health technology assessment (HTA)	30%
Speak publicly about a NEN-related policy issue	30%
Publish data	30%
Support academic institutions by funding fellowship programs, travel grants	15%
Provide specialist nurse support/services	10%
Provide professional psychological support	10%

It was also of note in the survey that 75% of organizations had seen activity engagement increase over the last 2 years, but of concern was the 20% for whose activities had decreased due to lack of funding, the global pandemic, the loss of critical staff, and issues in engaging volunteers (Table 1).

Evidence-based advocacy, i.e., advocating in a targeted, evidence-based, well-educated, and professional manner, and measuring impact and outcomes of what we do [10•], is of paramount importance, and collecting real-life data from the community provides tools to drive change in both commissioning for NEN treatments and clinical practice. Our advocacy roles are focused on having true impact within a community unaware of the needs of these patients. In the NEN community, many patient organizations fulfill that remit through a number of initiatives, surveys, research, and publications looking at patient priorities, e.g., optimizing treatment pathways [11], quality of life and supportive care needs [12, 13], financial toxicity implications [14••], and the burden of living with NENs [15••, 16••, 17].

By sharing knowledge and patient experience, we can collaboratively create positive change. NEN patient organizations work to enhance awareness and self-confidence among NEN patients so they can make informed choices and ask questions. It is vital that the patient voice helps facilitate best practice, and that alongside having an input into drug research and development, there is also a safe platform for sharing experiences and peer support. “Current data suggest that meaningful patient inclusion can help drive discussion and knowledge dissemination at academic medical conferences and widen research agendas to include new patient centered domains” [18].

## Challenges and Developments

In its capacity of a global umbrella organization, INCA is trying to focus the efforts of all its members on addressing the unmet needs in NENs, which in many cases can prove efficient only if driven on local level. In order to identify perceived unmet needs in the management of NENs from the perspectives of patients, patient advocates, and HCPs, INCA championed an international survey in 2017, which revealed significant gaps in the quality of information about the disease, access to diagnostics, treatment, and research. The results of this survey formulated important conclusions regarding patients’ information needs, access to optimal care, and involvement in research, and these have been presented at all major oncology and NEN-specific conferences, notably winning best poster awards at ESMO Congress 2018 and ENETS Conference 2019. An article based on the survey was published as an open-access manuscript in the International Journal of Cancer, which was in turn recognized as one of the top downloaded papers for the 2018–2019 period.

Equitable access to innovative diagnostic and treatment tools in NENs is a significant global challenge, and a focused effort should be directed at advocating to include NENs in national cancer plans. INCA strongly believes that public funding should be provided to the advocacy community to support their vital work in bridging the gap in provision of information [19••].

Research suggests that delivery of consistent and appropriate standards of care in NENs may be suboptimal worldwide [20••, 21]. It is already an established practice that patient representatives are routinely consulted, to inform decision-making in the context of Health Technology Assessment.

A clear pathway should be established for NENs, directed and supported by national health systems, so that all patients can reliably access a consistent standard of care. Healthcare systems should also aim to create more specialist centers with a focus on improving NEN patient outcomes. [19••] Almost a third (30%) of NEN patients had to travel more than 300 km/186 miles for treatment or consultation with a NET specialist, while 34% of patients did not have access to an MDT and those that did may be in contact with them less than once per year (14%). [22••] It is a must that patients with rare and less common cancers/diseases are treated in centers of expertise able to provide expert diagnosis, treatment, and continuity of care, including psychological and transition support. Only in this way can the clinical advances of the last two and half decades be built upon further to ensure that the care of these complex, lifelong patients can be considered truly holistic [23•].

There is an urgent need to increase awareness and specialized education in NENs among all relevant HCPs, as this may facilitate faster detection, diagnosis, and referral for patients [19••]. Data from the Survey of Challenges in Access to Diagnostics and Treatments for Neuroendocrine Tumor (NET) Patients (SCAN) show that almost half (46% [1077/2359]) of patients had stage IV NENs. Mean time to diagnosis was 5 years and 1042 were misdiagnosed, lower in Asia and higher in NA (all: Europe 4.05 vs. NA 6.44 vs. Asia 2.28 vs. Oceania 4.81 (*T*-test, *p*<0.0001) [24••].

The UK NET Patient Experience Survey reported that NEN patients were less likely to be given enough information about their condition and treatment compared with other cancer patients (71% vs. 88%) [25]. Other studies have also reported that for patients with more prevalent cancers, satisfaction and accessibility relating to hospitals, diagnostic, and therapeutic tools is superior and travel time shorter than for those with rarer cancers [26, 27].

The Unmet Needs Survey article identified many action points, with the following regarded as requiring urgent attention:

1. Improve utilization of existing resources, such as providing written information to patients at relevant stages of their pathway [19••]. While HCPs felt they provided patients with sufficient information (59%), informational needs were mostly or fully met for only 30% of patients and 18% of advocates [19••].

One of INCA’s major projects is the development of a Global NET Patient Information Pack, which is available in 10 languages and provides fact sheets covering signs/symptoms, tests, treatments, and supportive care to address the wrap-around care needs of NEN patients. Completion is expected in 2021.

2) Increase patient involvement in research by providing information about ongoing trials and facilitate access [19••]. The SCAN data complements this conclusion, showing that only 17% of NEN patients globally have already participated in clinical trials, and 79% would like to have more information on clinical trials, while 67% are willing to participate.
3) Conduct further research into NENs, particularly to put NEN research on equal footing with other major cancers, ensure earlier diagnosis, and to improve current treatments [19••].
4) Education of HCPs, this is vital to address the devastating late diagnosis that is so prevalent in our community [19••].
5) Support and development of specialized NEN clinics and MDTs equitably across the globe [19••].

In this context, patient organizations have an instrumental role as providers of education to patients and their families, as well as HCPs, so that both parties can engage in a meaningful and constructive dialogue. This is especially relevant in a setting of less common and not well-understood diseases where clear pathways and guidelines are still a challenge in many instances. In this respect, the NEN community is notably well-positioned to address such challenges in the spirit of collaboration.

## Future Directions

INCA has realized the crucial importance of multi-stakeholder collaboration, which has in fact been the true driver of its progress over the years. INCA is not a standalone organization because in order to be truly effective, it is essential to have adequate support and buy-in, as well as to continually instigate community engagement. Patient organizations especially in the rarer cancer field also have the added challenges of promoting disease awareness in an environment often not ready for the nuances of different cancers and obtaining funds to support research. There is no progress without research but there has to be fair distribution of research funds across the cancer spectrum.

Knowing that other communities share similar challenges, we needed to develop strategic partnerships across the wider cancer advocacy and rare disease community. To date, we engage closely with the International Union for Cancer Control (UICC), which collaborates with the WHO and UN, as well as regulatory bodies, the European Cancer Organisation, EURACAN—the European Reference Network for rare or low prevalence complex diseases, the Workgroup of European Cancer patient Advocacy Networks (WECAN), the National Organization for Rare Diseases (NORD), and the European Organisation for Rare Diseases (EURORDIS).

**Fig. 2:**
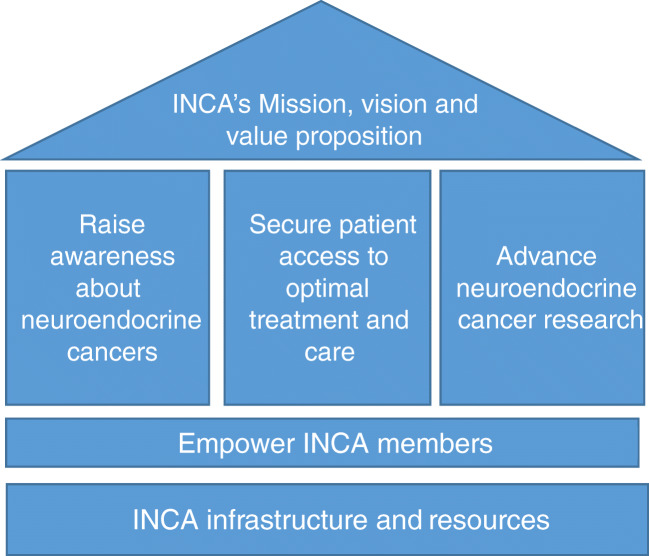
INCA values architecture

As an umbrella organization working to join the efforts of NEN patient advocacy groups around the globe, INCA has a clear directive moving forwards under its key strategic pillars of awareness/education, access, and research. (Fig. 2)

### Research

Key goals under the research pillar of activity are to help drive the NEN research agenda and push for research that will have the largest potential impact on transforming patient outcomes. To achieve these, INCA is collaborating closely with the NEN medical societies around the world: ENETS (European Neuroendocrine Tumor Society), NANETS (North American Neuroendocrine Tumor Society), APNETS (Asia-Pacific Neuroendocrine Society), CommNETs (Commonwealth Neuroendocrine Tumor Group), JNETS (Japan Neuroendocrine Tumor Society), CSNET (Chinese Neuroendocrine Tumor Society), among others.

The global NEN patient community has also identified as its priority the provision of patient-friendly information about ongoing research. Facilitating consensus among key stakeholders on the potential barriers to improving patient outcomes and research strategies that need to be deployed to advance research in the interest of patients is another major focus. Recognizing the significant value of informed/expert patient input into defining the global research agenda for NENs, INCA developed a training program to educate the community about research and their place in that area of medicine, a vital output for INCA. The INCA Boot Camp commenced in June 2020 to address a need of more active patient involvement in research, which is recognized among many stakeholders.

“The impact of involvement in individual research projects where researchers collaborate with patients/the public might be more usefully conceived as a form of experiential knowledge, expertise that is gained through the researchers’ direct experience of working with patients/the public. With this understanding, researchers’ accounts of involvement provide a source of insight and learning that might usefully inform the approaches used by others, in the same way that insights and learning from the patient experience can usefully shape research processes” [28].

The NEN patient community aspires to see patients involved in the following areas of the research pathways: protocol design, patient information development, regulatory and HTA committees, and post-study communication.

### Awareness and Education

Raising awareness and improving knowledge of NENs is definitely under the remit of the global NEN patient profile. Education at training level, as well as continuing medical education of healthcare professionals, clearly transpires as a priority. Increased awareness of NEN diagnostics and treatments, particularly newer, more specialized tools, among both HCPs and patients is required to ensure continued advancements and improvements in the global standard of care for NENs [29]. Looking at the SCAN results, the most common recommendations to improve NEN diagnosis and management globally were “more healthcare professionals knowledgeable in NETs” [30].

Ongoing provision of medically vetted, patient-friendly information on all types of NENs and the various aspects of living with the disease is done on a daily basis via various channels, both on local and global level. Last but not least, navigating patients and their families down the complicated and challenging pathway to timely diagnosis, care, and treatment is essential for improving patient experience and outcomes.

### Access

Strategic priorities needed to be set to drive success for patient access to optimal care and treatment. This started with mapping the extent of access challenges globally via the SCAN online survey, which was fielded during Sept–Nov 2019 and was available in 14 languages:

Arabic, Bulgarian, English, German, Dutch/Flemish, French, Japanese, Hindi, Italian, Mandarin (Chinese), Portuguese, Russian, Spanish, and Swahili. There were 2795 respondents from 68 countries: 2359 NEN patients/carers (Europe (47% [1102/2359]), North America (31% [727/2359]), Asia (12% [280/2359]), Oceania (9% [200/2359]), South America and Africa (2% [50/2359])) and 436 HCPs [29].

INCA has taken the results of this work to influence access at a global level and empower its members to address access challenges at a local level. These access issues are clearly visible even at the initial pathway stage of presenting symptoms and diagnosis with inappropriate diagnostic tools being used, leading to ineffective treatments. The consequence of this difficult pathway is a devastating late diagnosis for almost half of NEN patients around the world who had metastatic disease at diagnosis [29].

Hence, it is mandatory that the future path includes the following: development of well-defined cancer pathways, and the support for resource capability to ensure effective diagnostics and access to appropriate treatments for all. Centers of expertise are a must, certainly for the rare and less common cancers. One of the most common recommendations to improve NEN diagnosis and management given by NEN patients and healthcare professionals around the world is “better access to NET experts/specialist centers.” All cancer patients deserve to have access to a disease specialist for their particular cancer.

Alongside this, we also need to see a truly collaborative approach across all stakeholders, incorporating documented care needs of the patients. Should we not expand the gold standard MDT to incorporate the needs of the person affected by cancer rather than the healthcare system?

## Conclusion

Advocacy organizations are an integral part of the healthcare system and as such have a definitive role in supporting the strategic priorities in the neuroendocrine field: improving impact and outcomes for patients who follow a less common pathway, establishing patient experience as being on a par with clinical effectiveness, promoting the necessary investment to deliver a modern and high-quality service for patients (both in the acute and community setting), the provision of standardized useful and accurate patient information, a significant increase in the research, education, and awareness in NENs, ensuring commissioning processes are fit for purpose and that our community has the attention that is the right of all cancer patients. To achieve such priorities, a close and meaningful collaboration with the wider stakeholder communities, an understanding of the growth in the spectrum of what advocacy organizations can offer, and patient engagement are essential.

INCA operates on three clear levels to enable patients to raise their voice about this hidden cancer and promote a U-turn in the unmet needs that have been highlighted over many years:

1. Patient support: informing, supporting, and navigating.
2. Health policy: influencing health policy to ensure optimal access to diagnostics, treatment, and care.
3. Research: contributing in setting the research agenda in partnership with clinicians, academic networks, and industry.

The future is focused on the continuing development of advocacy organizations, keeping them visible and relevant, a natural part of the healthcare structure, not a separate entity. Collaborative working across the stakeholders in care, driven by advocacy organizations, will naturally bridge gaps between hospital care and living with cancer on a day-to-day basis at home.

The neuroendocrine community has struggled to get itself into the oncology arena. Raising awareness of the validity of this disease as a cancer and giving this cancer type equal attention is a right for any patient and their family. The ultimate goal is to get to a stage where our clinical pathway is as clear as our patient pathway, and a significant improvement in diagnostics times and standardization of care across the globe is witnessed.

Working towards that goal demands that NEN patient advocacy organizations continue gathering robust evidence, adapting to new environments and committing to expand their expertise. There is a precedence required in realizing the severity of the cancer type, understanding the broad spectrum of behaviors, the devastating impact the symptoms can have, and the dissolution of the idea that neuroendocrine neoplasms are less significant types of cancer. Removing that myth totally can only be achieved by sharing the experience of our patient community in a valid and statistically relevant way.

Ultimately, the NEN patient advocacy community aspires to remove restrictions to access, improve the atrocious time to diagnosis for so many, see resource capability and service delivery infrastructure for specialized centers across the globe, and to provide the patient voice to inform and educate all those involved in the care of neuroendocrine cancer patients. We can achieve this by working together, changing attitudes, and driving change.
